# Rosai dorfman disease of the orbit

**DOI:** 10.1186/1756-8722-1-7

**Published:** 2008-06-28

**Authors:** Geeta K Vemuganti, Milind N Naik, Santosh G Honavar

**Affiliations:** 1 Ophthalmic Pathology Service, Hyderbad Eye Research Centre, L V Prasad Eye Institute, Hyderbad, India; 2 Division of Ophthalmic Plastic Surgery, Orbit, and Ocular Oncology, LV Prasad Eye Institute, Hyderabad, India

## Abstract

**Objective:**

To report the clinico-histopathologic features, management and outcome of Rosai-Dorfman disease of the orbit.

**Design:**

Non-comparative case series.

**Results:**

Rosai-Dorfman disease of the orbit constituted 0.09% of all ocular specimens received at our Institute, presenting with a firm rubbery mass causing proptosis; bilateral in 4 (57%) cases. The median age at presentation was 13 years (range 5–65); median duration of symptoms was 6 (range 3–15) years. Lymphadenopathy was noted in 4 (57%); extranodal involvement in 3 (43%). After biopsy, 3 cases were treated with systemic corticosteroids, 2 cases developed local recurrence that responded to systemic corticosteroid therapy. Polymorphous population of lymphocytes, plasma cells, and characteristic S-100-positive histiocytes showing emperipolesis were pathognomonic histologic features.

**Conclusion:**

Rosai-Dorfman disease of the orbit, although rare, should be considered in young individuals with chronic proptosis with rubbery masses. Excision and corticosteroid therapy provide a favorable outcome.

## Background

Rosai-Dorfman disease, also known as sinus histiocytosis with massive lymphadenopathy is a rare disorder characterized by nonmalignant proliferation of distinctive histiocytes within lymph node sinuses and other extranodal sites. It is a self-limiting disorder of unknown etiology that occurs worldwide in children and young adults [1-5].

Classically, Rosai-Dorfman disease manifests as chronic painless cervical lymphadenopathy with pyrexia, leucocytosis, increased erythrocyte sedimentation rate and hypergammaglobulinemia. About 43% of patients have extranodal manifestation in the eye, upper respiratory tract, salivary gland, skin, bone, meninges and central nervous system and testis [3-5]. The reported ophthalmic manifestations include eyelid and orbital mass, and rarely uveitis [6-9]. Orbital involvement could be an isolated extranodal manifestation or associated with concurrent systemic disease. Orbital mass in Rosai-Dorman disease could mimic lymphoma, lacrimal gland tumors, and other histiocytic tumors. We herein report the clinical manifestations, management, and outcome of a series of seven histopathologically proven cases of Rosai-Dorfman disease of the orbit.

## Results

### Clinical Profile

Of the 7537 ocular specimens received during the study period, seven patients were diagnosed as Rosai-Dorfman disease, constituting 0.09 % of all ocular specimens and 2.3% of orbital lesions (7 of 300). The median age was 13 years (range 5–65), with male: female ratio of 3:4. Median duration of symptoms at presentation was 6 years (range, 3–15 years). The clinical profile of these patients is shown in [Additional File 1]. The presenting symptoms were painless progressive proptosis in all except two patients who presented with an eyelid mass. The referral diagnosis included orbital lymphoma, xanthogranuloma, and lacrimal gland tumor. Three patients had earlier undergone biopsy earlier elsewhere with an inconclusive diagnosis (slides not available for review).

**Figure 1:**
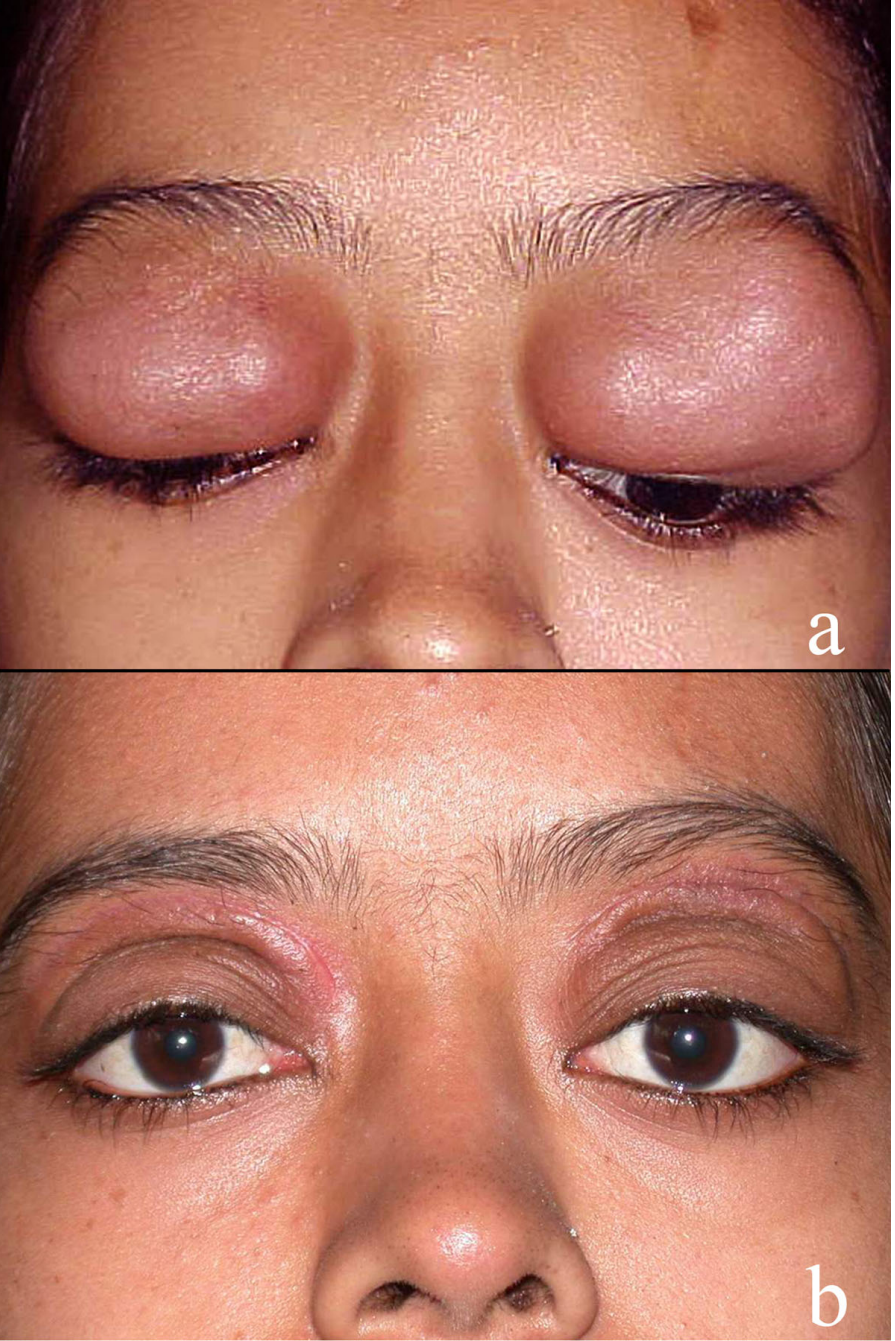
**Bilateral upper eyelid and preseptal orbital mass in a 16-year-old female with complete mechanical ptosis (1a). Published with permission from Elsevier**. This figure was published in the Clinical Ophthalmic Oncology, Vemuganti GK, Honavar SH, Eyelid Stroma Tumors, Page 105, Copyright Elsevier 2007. Three-month post-operative appearance following complete excision of the mass (1b).

At baseline evaluation, the visual acuity was 20/20 in all except one patient (patient 4) [Additional File 1] who had senile cataract. Diffuse conjunctival congestion was noted in 3. There was restricted ocular motility in 5 patients and mechanical ptosis in one. All patients had proptosis, which was unilateral in 3 (43%) and bilateral in 4 (57%). All patients had a palpable well-defined, nontender, rubbery, firm orbital mass with variable intrinsic mobility. The mass was preseptal in 2 patients (figure 1a), anterior orbital in 1, diffuse orbital in 3 and involved the orbital lobe of the lacrimal gland in 1. The preauricular, submandibular and anterior cervical lymph nodes were massive, confluent, nontender and rubbery in 4 patients. Computed tomography scans revealed a homogeneous soft tissue mass in a preseptal location in 2, in the anterior orbit in 2, diffuse in 2, and involving the lacrimal gland in 1.

Concurrent extranodal locations included the parotid gland in 1, paranasal sinus in 2, and nasopharynx in 1. Two patients had normocytic hypochromic anemia. The erythrocyte sedimentation rate was within the normal range in all patients. None had evidence of hepatosplenomegaly on clinical examination or imaging.

The primary management consisted of surgery in all cases. In three patients the preseptal and anterior orbital mass was well defined so as to allow easy dissection and separation from the surrounding structures; they underwent complete excision (figure 1b). Three patients with diffuse orbital mass and one with lacrimal gland involvement underwent an incisional biopsy (near total excision). Three patients with diffuse orbital mass received systemic corticosteroids at 1 mg/kg body weight initially and tapered over 6 weeks to 3 months. The mean duration of follow-up was 18.3 ± 17.7 months (range 0–80 months). Of six patients who had follow-up evaluation, two developed local recurrence. The first patient, who had undergone macroscopically apparent complete excision of a bilateral anterior orbital mass, developed unilateral diffuse orbital recurrence 2 months later. The second patient developed diffuse bilateral orbital recurrence and concurrent involvement of the maxillary sinus 10 months following complete excision of bilateral eyelid mass. Both these patients had not received systemic corticosteroid therapy as part of the primary treatment [Additional File 1]. The recurrence was treated with systemic corticosteroids. At the final follow-up, all patients had improvement in proptosis and cosmetic appearance. None had residual ocular motility restriction or ptosis.

### Histopathology

**Figure 2:**
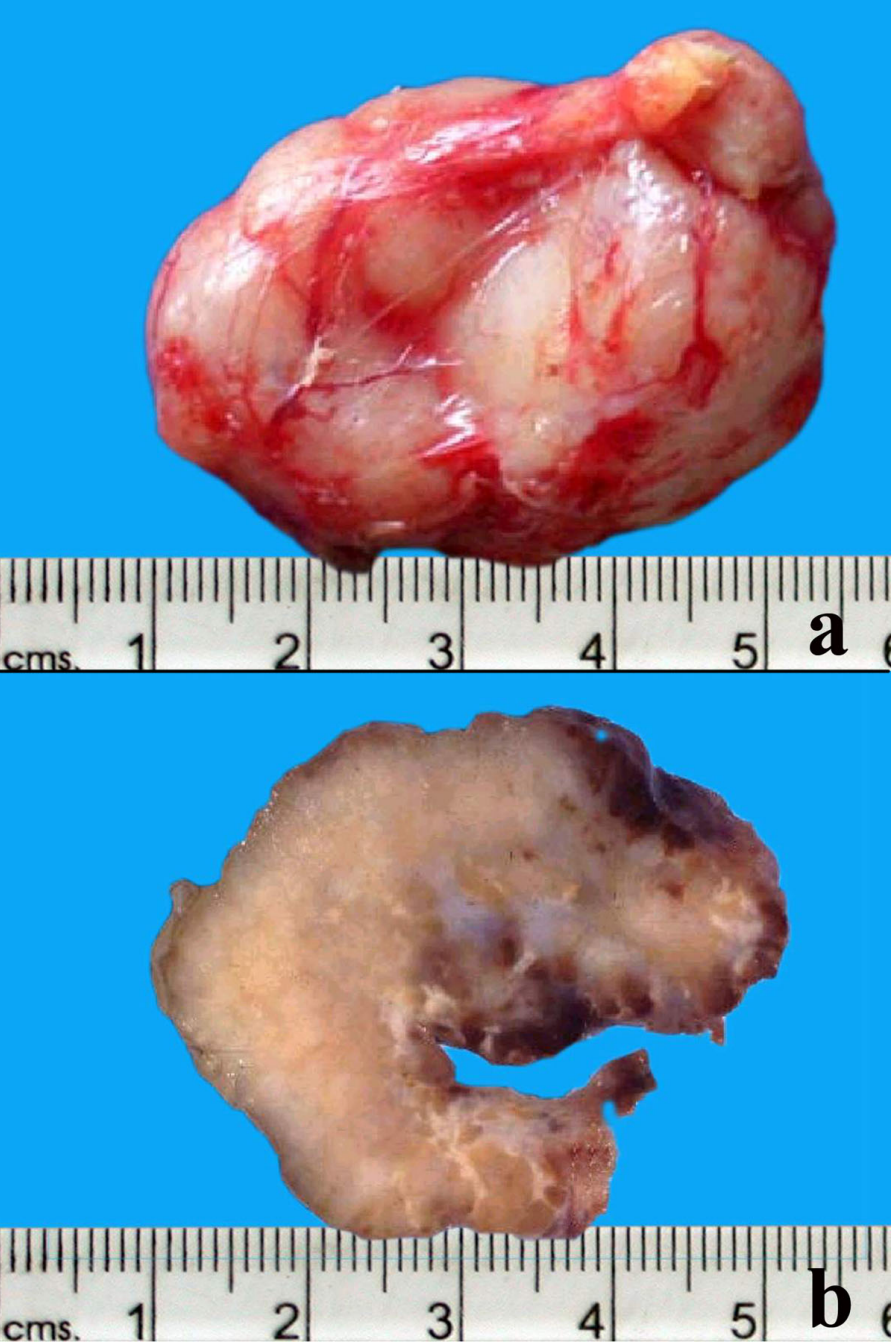
**The gross specimen of the excised mass with lobulated and smooth surface (2a)**. The cut section of the specimen shows a solid appearance with a few yellowish areas (2b).

**Figure 3:**
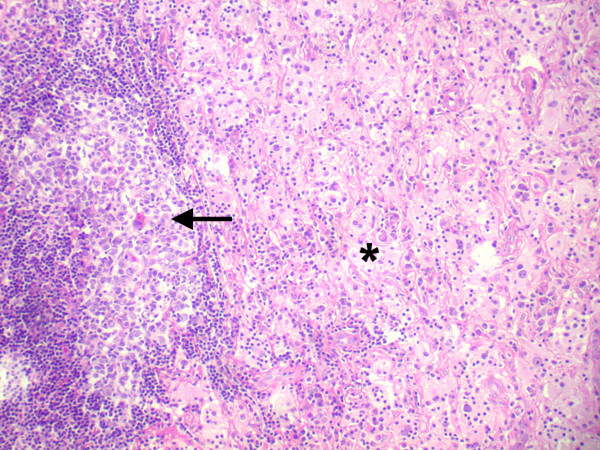
Histopathologic section showing a lymphoid follicle (arrow) surrounded by a cuff of lymphocytes and plasma cells with a few pale areas consisting of sheets of histiocytes (asterisk) (× 50, hematoxylin, eosin).

**Figure 4:**
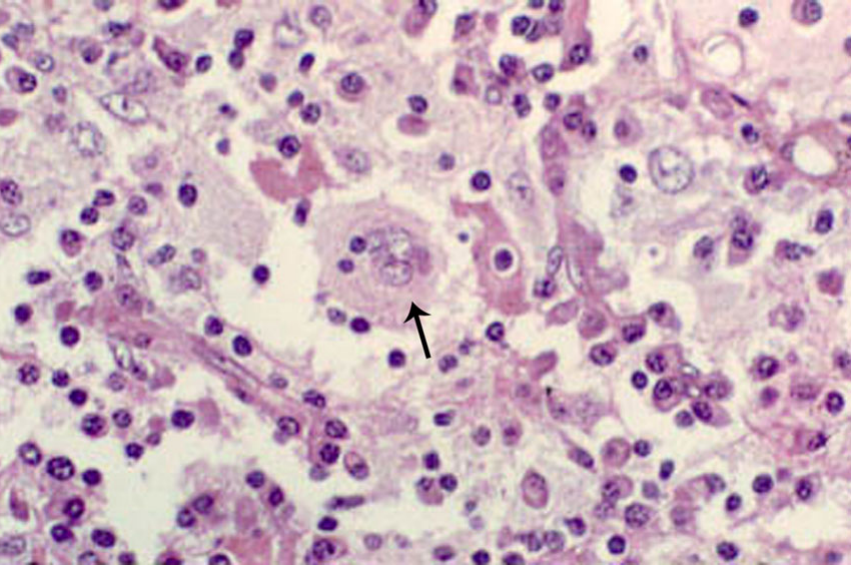
Histopathologic section showing a polymorphous population of cells consisting of mature lymphocytes and plasma cells interspersed with histiocytes. Histiocytes are large with a vesicular nuclei and abundant cytoplasm with engulfed lymphocytes and plasma cells, a phenomenon called lymphophagocytosis or emperipolesis, a hallmark of Rosai-Dorfman disease (arrow) (× 500, hematoxylin, eosin). Published with permission from Elsevier. This figure was published in the Clinical Ophthalmic Oncology, Vemuganti GK, Honavar SH, Eyelid Stroma Tumors, Page 105, Copyright Elsevier 2007.

**Figure 5:**
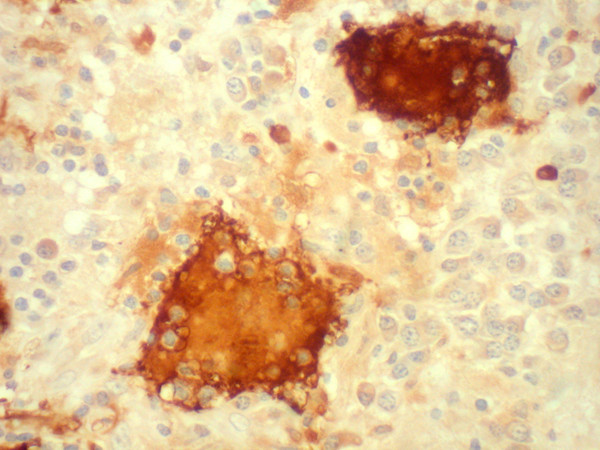
Histopathologic section showing large histiocytes with emperipolesis, immunoreactive for S-100 antigen. (× 200, DAB).

**Figure 6:**
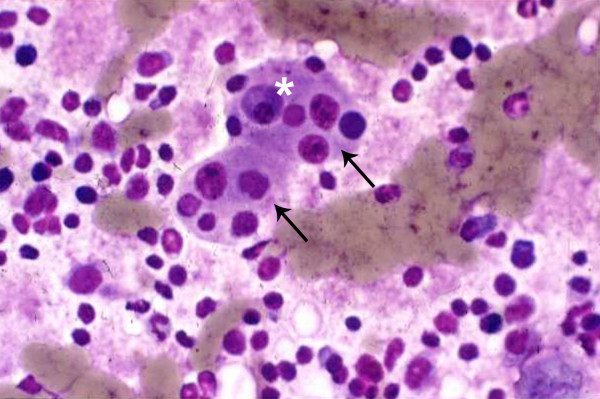
Imprint cytology shows large histiocytes (arrow) with vesicular nucleus and phagocytosed lymphocytes and plasma cells (asterisk) within the cytoplasm (× 500, Giemsa).

All lesions had similar histopathologic characteristics. On gross examination, the specimens measured 20 to 50 mm in dimension (figure 2a), was firm to rubbery in consistency with a lobulated surface. Cut sections revealed focal yellowish areas within the lesion (figure 2b). Microscopically, there was a polymorphous population of histiocytes, plasma cells and mature lymphocytes separated by fibrous septa (figure 3). A few lymphoid follicles were seen with germinal centers. In addition, there were prominent histiocytes which contained abundant pale to vacuolated cytoplasm and a large nucleus with prominent nucleoli. Many of these cells showed lymphophagocytosis or emperipolesis (figure 4). These histiocytes were immunoreactive to the S-100 antibody (figure 5). The plasma cells were seen clustered with extracellular and intracellular immunoglobulin deposits. Immunophenotyping revealed polyclonal population of plasma and lymphocytes. Imprint smears prepared from the 4 unfixed fresh specimens available, revealed characteristic histiocytes with emperipolesis in a background of plasma cells and lymphocytes (figure 6).

## Discussion

In 1969, two pathologists Juan Rosai and Ronald Dorfman reported a distinct histiocytic disorder in young black males presenting with bilateral, painless, massive cervical lymphadenopathy with a protracted clinical course, and in most instances associated with fever, anemia, neutrophilia, elevated erythrocyte sedimentation rate, and polyclonal gammopathy[1]. The clinicopathological conglomerate that they named sinus histiocytosis with massive lyphadenopathy has now come to be known as Rosai-Dorfman disease[1]. Subsequently it became clear that the disease had no specific predilection for geographic location or race [5].

Painless lymphadenopathy is the most frequent systemic presenting symptom and involves the cervical region in up to 90% of patients [5]. Other locations such as inguinal (26%), axillary (24%) and mediastinal lymph nodes (15%) are also reported to be involved [5].

Extranodal disease is documented in 43% of patients, in some without associated lymphadenopathy, which may or may not develop later in the disease course [5]. The most common extranodal sites, in the decreasing order of frequency, are skin, nasal cavity and paranasal sinus, eyelid, orbit, bone, salivary gland and central nervous system [Additional File 2] [5]. The simultaneous involvement of multiple extranodal sites is not unusual [5]. Hepatosplenomegaly, unlike in other histiocytic disorders, is uncommon [5].

The high prevalence of this disease in this series (2.3% of orbital lesions and 0.09% of ocular specimens) is possibly because this center is a tertiary eye referral center with a dedicated Ocular Oncology Service. With increasing number of ocular specimens received at our centre to nearly 4,000 samples per year, the prevalence may now match the same as seen in any general hospital, i.e 0.03% [5/15,000 cases based on the personal communication received from a surgical pathologist ]. The reported ophthalmic manifestations of Rosai-Dorfman disease include orbital and eyelid involvement, lacrimal gland involvement, optic nerve compressive neuropathy and uveitis [3,6-12]. Orbital involvement is the most common of ophthalmic manifestations [3,6-12]. While a majority of patients with orbital involvement have concurrent lymphadenopathy, some may present with orbit as the sole extranodal site of involvement without synchronous nodal disease, and a minority may have concurrent involvement of other extranodal sites such as the paranasal sinus [3]. In our series of seven patients, six were under 20 years of age and had chronic symptoms ranging from 3–15 years. All had painless progressive proptosis and two had an eyelid mass. Four patients had bilateral manifestations, 4 had synchronous nodal disease and 3 had concurrent extranodal involvement.

Clinical laboratory findings in Rosai-Dorfman disease include hematological abnormalities such as normocytic or microcytic anemia, hemolytic anemia, elevated erthrocyte sedimentation rate and polyclonal hypergammaglobulinemia [5]. Two patients in our series had normocytic hypochromic anemia. However, erythrocyte sedimentation rate was within the normal range in all patients and serum electrophoresis did not reveal hypergammaglobulinemia.

According to the Writing Group of the Histiocyte Society [13], the histiocytic syndromes can be subdivided based on whether the proliferating histiocytes are the Langerhans cells or not and whether the process is benign or malignant. Rosai-Dorfman disease is one of the non-Langerhans cell benign histiocytosis where predominantly the sinuses of the lymph nodes, and less commonly the interfollicular area of the lymph nodes are infiltrated with distinctive histiocytes with round or oval vesicular nuclei with well-defined, delicate nuclear membranes and a single prominent nucleolus [1,2,5]. Nuclear atypia and mitoses are infrequent. The hallmark of Rosai-Dorman disease is lymphophagocytosis or emperipolesis, wherein the viable lymphocytes are located in well-defined cytoplasmic vacuoles of intact histiocytes. Plasma cells, neutrophils and red blood cells may also occupy this unique intracytoplasmic niche. The involved histiocytes are activated macrophages with features of phagocytic cells as well as immune accessory cells and thus express S-100 protein, HAM 56, α1 antitrypisn, α1 chymotrypsin, lysozyme, Mac 387, Ki-1 (CD 30, Ber-H2), but are negative for CD 1a (leu 6) [14]. Rosai-Dorfman disease involving extranodal sites shows similar morphologic features to its nodal counterpart with more fibrosis and fewer histiocytes with emperipolesis. The histological differential diagnosis includes hemophagocytic syndromes, storage disorder, inflammatory lesions, necrobiotic xanthogranuloma and lymphoreticular malignancies [15]. The presence of benign histiocytes with emperipolesis, absence of cellular atypia, immunohistochemical profile, and associated clinical features distinguish Rosai-Dorfman disease from other simulating disorders.

Cytologic features of Rosai-Dorfman syndrome are well recognized and the role of fine-needle aspiration cytology in its diagnosis has been demonstrated [16,17]. We used the impression cytology technique for rapid intraoperative diagnosis of Rosai-Dorfman disease based on known cytological characteristics in 4 patients in this series. The cytologic diagnosis correlated with the final histopathology in all four patients.

Despite its well-recognized clinical presentation, the precise etiology of Rosai-Dorfman disease remains unknown. The etiologic factors implied in the pathogenesis of this disease are bacterial (Klebsiella), virus (Epstein barr virus, parvovirus B 19), immune dysfunction, or an aberrant response to an unspecified antigen, HHV-6 or EBV [18-21]. Current thinking is that the defective Fas/FasL signaling leading to altered apoptosis may be an important mechanism whereby uncontrolled histiocytic proliferation is triggered [20]. The presence of characteristic histiocyte, derived from circulating mononuclear cells, long history and an increased incidence of serum autoreactive antibodies during active disease suggest a possible pathogenic correlations with a dysregulatory process. In a recent report, the evidence points towards a viral etiology as suggested by the immunolocalization of parvovirus B19 (B19) virus using antibodies against B19 capsid proteins VP1/VP2[21]. The relative increase of cases from this part of the world, also prompts us to believe that there could be a possible environmental factor, thereby warranting further studies in this direction. Though not done in this series, immunological studies, specifically for viral etiology, liver function along with follow-up to identify known risk factors like airway compression, would be beneficial in understanding more about this rare disease [21,22].

The clinical course of Rosai-Dorfman disease is chronic and variable with episodes of exacerbation alternating with periods of remission, where the timing and duration of each phase is entirely unpredictable. Foucar et al reported stable disease in 54%, spontaneous regression in 21%, and progressive disease in only 1% [5].

The ideal treatment for Rosai-Dorfman disease is yet unestablished. Only about 50% of patients with Rosai-Dorfman disease need some form of treatment [20]. Management options include observation for mild manifestations with no cosmetic or functional abnormality, surgical excision or debulking for lesions in surgically accessible locations, and systemic corticosteroids, chemotherapy or radiotherapy in patients with severe symptoms where vital organ function is compromised [23-27]. Radiotherapy and antimetabolite treatment has been considered in a few cases but the literature review by Pulsoni et al does not suggest any conclusive role of these treatment modalities [27]. The treatment of orbital manifestations of Rosai-Dorfman disease aims to control the functional and cosmetic abnormalities. Orbital involvement, being cosmetically disturbing and surgically accessible, may be more often considered for surgical treatment. Massive or recurrent orbital disease or significant residual lesion following surgical debulking may be treated with systemic corticosteroids, chemotherapy or radiotherapy. Chemotherapy has also been used to relieve the sight threatening optic nerve compression [10].

All patients in our series underwent surgery – 3 with well-defined localized mass underwent surgical excision and 3 with diffuse orbital involvement and 1 with lacrimal gland involvement underwent an incisional biopsy. Three patients with diffuse orbital involvement received systemic corticosteroid therapy. Local recurrence was noted in 2 of 7 (29%) cases, both within one year of primary treatment, and these patients responded to systemic corticosteroids.

## Conclusion

To conclude, Rosai-Dorfman disease may be suspected in young individuals with unilateral or bilateral slowly progressive proptosis manifesting with a rubbery firm mass, with or without massive cervical lymphadenopathy. Systemic evaluation is necessary to document other extranodal sites of involvement. A biopsy will help confirm the diagnosis. Patients with cosmetic and/or functional abnormality secondary to the orbital mass could be considered for debulking or complete excision. Diffuse, residual, or recurrent lesions may be treated with systemic corticosteroids.

## Methods

We reviewed consecutive cases of orbital tumors on the registry of the Ophthalmic Pathology Service at a tertiary care centre between January 1996 and December 2002 and included patients with a histopathologic diagnosis of Rosai-Dorfman disease in this series. The medical records of these patients were reviewed for the demographic data, clinical manifestations and radiologic features, management and outcome.

An experienced ophthalmic pathologist reviewed formalin-fixed, paraffin-embedded hematoxylin-eosin stained sections. Immunohistochemistry was performed on the sections using S-100 protein and monoclonal antibodies against T-and B-cell markers, and lambda and kappa chains of immunoglobulins.

## Competing interests

The authors declare that they have no competing interests.

## Authors' contributions

GKV conceived the idea, carried out the histopathologic studies, drafted the manuscript and reviewed the review of literature, MN participated in the study design, and reviewed the cases, SGH participated in the study design and provided critical inputs into the study. All authors read and approved the final manuscript

## Consent

The patients have given their consent for the medical records to be reviewed for research and publications through the informed consent.

## Supplementary Material

Additional file 1 Table 1 Clinical features of seven patients with Rosai-Dorfman disease of the orbit. This table describes the clinical features of all cases of Rosai Dorfman Disease of the orbit included in this study. Click here for file

Additional file 2 Table 2: Clinical manifestations of Rosai-Dorfman disease. This table describes the site, frequency and the clinical manifestation of Rosai Dorfman Disease. This research was originally published in *Blood*. McClain KL, Natkunam Y, Swerdlow SH. Atypical cellular disorders. *Blood*. 2004;:283–96. ^© ^American Society of Hematology. Click here for file
