# Supplementary material for: Rosai dorfman disease of the orbit
Source: J Hematol Oncol. 2008 Jun 28;1:7. doi: 10.1186/1756-8722-1-7 (PMC2474646; doi:10.1186/1756-8722-1-7)
Supplement: Additional file 1 — Table 1 Clinical features of seven patients with Rosai-Dorfman disease of the orbit. This table describes the clinical features of all cases of Rosai Dorfman Disease of the orbit included in this study. [file 1756-8722-1-7-S1.doc]

Table 1. Clinical features of seven patients with Rosai-Dorfman disease of the orbit

| **Number** | **Age/ Gender** | **Laterality** | **Duration**  **of**  **Symptoms**  **(Years)** | **Location** | **Lymph Node Involvement** | **Other Extranodal Sites** | **Primary Treatment of the Orbital Lesion** | **Recurrence of the Orbital Lesion** | **Treatment of Recurrence of the Orbital Lesion** | **Duration**  **of**  **Follow-up**  **(Months)** |
| --- | --- | --- | --- | --- | --- | --- | --- | --- | --- | --- |
| 1 | 5/M | Bilateral | 3 | Eyelid, Preseptal Orbit | No | None | Surgical excision | No | NA | 0 |
| 2 | 16/F | Bilateral | 6 | Eyelid,  Preseptal Orbit | Yes | Paranasal sinus | Surgical excision | Yes | Systemic corticosteroids | 18 |
| 3 | 18/F | Bilateral | 15 | Anterior Orbit | Yes | Parotid gland | Surgical excision | Yes | Systemic corticosteroids | 80 |
| 4 | 65/F | Unilateral | 3 | Lacrimal gland | No | None | Surgical Excision | No | NA | 3 |
| 5 | 13/M | Unilateral | 4 | Diffuse Orbit | Yes | None | Incisional Biopsy + Systemic corticosteroids | No | NA | 12 |
| 6 | 13/M | Unilateral | 7 | Diffuse Orbit | No | Paranasal sinus, Nasopharynx | Incisional Biopsy + Systemic corticosteroids | No | NA | 12 |
| 7 | 10/F | Bilateral | 9 | Diffuse Orbit | Yes | None | Incisional Biopsy + Systemic corticosteroids | No | NA | 3 |
