# Supplementary material for: Rosai dorfman disease of the orbit
Source: J Hematol Oncol. 2008 Jun 28;1:7. doi: 10.1186/1756-8722-1-7 (PMC2474646; doi:10.1186/1756-8722-1-7)
Supplement: Additional file 2 — Table 2: Clinical manifestations of Rosai-Dorfman disease. This table describes the site, frequency and the clinical manifestation of Rosai Dorfman Disease. This research was originally published in Blood. McClain KL, Natkunam Y, Swerdlow SH. Atypical cellular disorders. Blood. 2004;:283–96. © American Society of Hematology. [file 1756-8722-1-7-S2.doc]

Table 2. Clinical manifestations of Rosai-Dorfman disease

| **Anatomic Location** | **Frequency^5^** | **Clinical Manifestation** |
| --- | --- | --- |
| Lymph nodes | 87% | Massive painless lymphadenopathy, unilateral or bilateral, most commonly affecting cervical lymph nodes |
| Skin and Soft Tissue | 16% | Cutaneous lesions (maculopapular rash, xantomatous lesion, reddish or bluish mass), or subcutaneous nodules |
| Nasal cavity and Paranasal sinus | 16% | Mucosal thickening, polyp, infiltrative paranasal sinus mass |
| Eye and adnexa | 11% | Orbital or eyelid mass |
| Bone | 11% | Osteolytic lesions of axial or appendicular skeleton with or without sclerosis |
| Salivary gland | 7% | Bilateral parotid or submadibular gland mass |
| Central nervous system | 7% | Intracranial, epidura, dural or spinal mass |
| Oral cavity | 4% | Infiltrative mass of soft or hard palate, diffuse mucosal thickening, papillomatosis or gingival hypertrophy |
| Kidney and Genitourinary tract | 3% | Renal mass with urinary obstruction, testicular, scrotal or ependymal mass, penile edema |
| Respiratory tract | 3% | Laryngeal obstruction, vocal cord thickening with hoarseness of voice |
| Liver | 1% | Hepatomegaly, military infiltration, mass |
| Tonsil | 1% | Enlargement |
| Breast | <1% | Subcutaneous nodules |
| Gastrointestinal tract | <1% | Mucosal hypertrophy, mass |
| Heart | <1% | Subendocardial and valvular infiltrates casing arrythmia |
